# Supplementary material for: Health-Related Content of TV and Radio Advertising of Dietary Supplements—Analysis of Legal Aspects after Introduction of Self-Regulation for Advertising of These Products in Poland
Source: Int J Environ Res Public Health. 2022 Jun 30;19(13):8037. doi: 10.3390/ijerph19138037 (PMC9265686; doi:10.3390/ijerph19138037)
Supplement: Supplementary file 1 [file ijerph-19-08037-s001.zip › ijerph-1737299-supplementary.pdf]

Table S1. Działanie suplementów diety lub ich składników w świetle reklamy.

DZIAŁANIE SUPLEMENTÓW DIETY
**Rodzaj informacji**
**Część A.**<br>**Nierzetelny przekaz zapewniający o działaniu w organizmie**<br>(niezgodny z rozporządzeniem (UE) Nr 1169/2011, rozporządzeniem (WE) Nr 1924/2006 i Porozumieniem Nadawców)
**1.** *12 tygodni i efekt jak widać (na ekranie TV wizerunek wysportowanych osób, wskazujących na sprawność układu kostno-stawowego)*<br>**2.** *Jak stosuję (nazwa suplementu diety) nie obawiam się już uderzeń gorąca, wahań nastroju, zimnych potów i nadmiernej masy ciała*<br>**3.** *(nazwa suplementu diety)… i pamiętam o wszystkim. Stosuję, aby pamięć działała bez szwanku*<br>**4.** *Kilogramy giną bezpowrotnie, skuteczne odchudzanie*<br>**5.** *Na odchudzanie - sprawdzony, skuteczny*<br>**6.** *Z... (nazwa suplementu) zapomniałam o cellulicie*<br>**7.** *Uspokoisz się, wyśpisz się*<br>**8.** *(nazwa suplementu) …to zdrowa wątroba plus masa ciała przy okazji*<br>**9.** *Mnie pomógł (nazwa suplementu). Problem z trzymaniem moczu - mam już to za sobą*<br>**10.** *(nazwa suplementu) …to dla mnie spokojnie przespana noc*<br>**11.** *Twoje włosy stają się gęste, lśniące i zdrowe*
**Część B.**<br>**Przekaz o działaniu wspomagającym organizm**
**1.** *Wspomaga odporność*<br>**2.** *Sposób na odporność*<br>**3.** *Pozwoli ci zadbać o odporność*<br>**4.** *Na problemy z apetytem, wspiera apetyt, ułatwia trawienie*<br>**5.** *Wspomaga trawienie i redukcję nadmiaru gazów, wspiera pracę żołądka, wątroby i jelit*<br>**6.** *Wspiera produkcję kolagenu*<br>**7.** *Dla dobra stawów*<br>**8.** *Dla mocnych, zdrowych kości*<br>**9.** *Osłania gardło*<br>**10.** *Dba o mikrobiom*<br>**11.** *Łagodzi objawy menopauzy*<br>**12.** *Pełne wsparcie dla oczu*<br>**13.** *Wspiera prawidłowe widzenie*<br>**14.** *Pomaga mi utrzymać wagę*<br>**15.** *Ułatwia szybsze zasypianie*<br>**16.** *Wspomaga nieprzerwany sen i daje energię*<br>**17.** *Pomaga radzić sobie ze stresem*<br>**18.** *Sposób na odchudzanie*<br>**19.** *Wspiera kompleksowo*
**DZIAŁANIE SKŁADNIKÓW SUPLEMENTÓW DIETY**
**Rodzaj informacji**
**Część C.**<br>**Nierzetelny przekaz zapewniający o działaniu w organizmie**

(niezgodny z rozporządzeniem (UE) Nr 1169/2011, rozporządzeniem (EC) Nr 1924/2006 i Porozumieniem Nadawców)
**1.** *Wyciąg z ostryżu długiego zapobiega gromadzeniu się tłuszczu*<br>**2.** *Wyciągi z ziół skoncentrowane na trawieniu, które uwalniają od uczucia ciężkości*<br>**3.** *Składniki aktywne utrzymują prawidłowe napięcie mięśni, w tym mięśni mających wpływ na trzymanie moczu*
**Część D.**<br>**Nierzetelny przekaz o działaniu wspomagającym organizm**
**1**. *Cynk pozytywnie wpływa na zmęczone oczy* (niezgodny z rozporządzeniem (EC) Nr 432/2012)<br>**2.** *Wyciąg z Cola nitida wspiera intensywne spalanie tłuszczu, pomagając w skutecznej redukcji wagi* (niezgodny z rozporządzeniem (EC) Nr 1924/2006)
**Część E.**<br>**Pozostałe oświadczenia o działaniu wspomagającym organizm**
**1.** *Witamina D pomaga w prawidłowym funkcjonowaniu układu odpornościowego*<br>**2.** *Witamina D pomaga utrzymać zdrowe kości, zęby i mięśnie*<br>**3.** *Witamina D jest niezbędna dla zdrowych kości*<br>**4.** *Witamina D wspiera wchłanianie wapnia*<br>**5.** *Witamina D wspiera dzieci w ich rozwoju*<br>**6.** *Witamina D bardzo istotna dla odporności*<br>**7.** *Witamina D wspiera odporność*<br>**8.** *Witamina C pomaga w prawidłowej produkcji kolagenu, w celu zapewnienia prawidłowego funkcjonowania skóry i chrząstki*<br>**9.** *Witamina C wspiera prawidłowe funkcjonowanie układu odpornościowego*<br>**10.** *Witamina A pomaga w prawidłowym funkcjonowaniu układu odpornościowego*<br>**11.** *Witamina A pomaga zachować zdrową skórę*<br>**12.** *Witamina A wspiera odporność*<br>**13.** *Witamina K_2 dla zdrowych kości*<br>**14.** *Biotyna pomaga zachować zdrowe włosy i skórę*<br>**15.** *Biotyna pomaga w utrzymaniu prawidłowych funkcji psychologicznych*<br>**16.** *Cynk pomaga w utrzymaniu prawidłowego widzenia*<br>**17.** *Magnez pomaga w prawidłowym funkcjonowaniu układu nerwowego*<br>**18.** *Wapń pomaga utrzymać zdrowe kości, zęby i mięśnie*<br>**19.** *Kwasy tłuszczowe omega-3 przyczyniają się do prawidłowego funkcjonowania serca*<br>**20.** *Kwas DHA wspomaga prawidłowe funkcjonowanie mózgu dziecka*<br>**21.** *Melatonina ułatwia zasypianie*<br>**22.** *Czosnek wspiera zdolność antyoksydacyjną organizmu i naturalne mechanizmy obronne*<br>**23.** *Czosnek wspomaga mechanizmy obronne oraz pomaga utrzymać zdrowy układ oddechowy*<br>**24.** *Wyciąg z dzikiej róży wspiera odporność*<br>**25.** *Wyciąg z mięty pieprzowej wspomaga trawienie i prawidłowe funkcjonowanie przewodu pokarmowego oraz pomaga zachować zdrowy żołądek*<br>**26.** *Wyciąg z koniczyny pomaga łagodzić objawy menopauzy*<br>**27.** *Wyciąg z szyszek chmielu pomaga łagodzić objawy menopauzy*<br>**28.** *Wyciąg z szyszek chmielu przyczynia się do spokojnego snu,*<br>**29.** *Pieprz kajeński przyczynia się do redukcji masy ciała*<br>**30.** *Ostrokrzew paragwajski pomaga w utrzymaniu prawidłowej wagi*<br>**31.** *Wyciąg z liści karczocha wspiera detoksykację, stymuluje wydzielanie soków trawiennych oraz pomaga w utrzymaniu zdrowej wątroby, przyczynia się do komfortu jelitowego*<br>**32.** *Wyciąg z owoców kopru wspiera trawienie, eliminację nadmiaru gazów oraz pomaga w prawidłowym trawieniu tłuszczów*<br>**33.** *Wyciąg z ostryżu długiego pomaga w utrzymaniu prawidłowego funkcjonowania wątroby*

34. *Wyciąg z krokusa uprawnego przyczynia się do równowagi emocjonalnej*
35. *Wyciąg z nasion winogron pomaga w utrzymaniu prawidłowej masy ciała i zmniejszeniu cellulitu*
36. *Karczoch, rozmaryn, kurkuma przyczyniają się do wspomagania trawienia oraz komfortu trawiennego*
37. *Skrzyp polny pomaga w zachowaniu zdrowych włosów, skóry i paznokci*
38. *Len wspiera kontrolowanie wagi*
39. *Aminokwasy budujące keratynę naturalnie występującą we włosach i rzęsach*
40. *Składniki pomagają w utrzymaniu koncentracji i sprawności umysłowej*
41. *Składniki łagodzą uderzenia gorąca i nerwowość, wspomagają spokojny sen i młody wygląd skóry*
